# Stuck in limbo: illicit drug users’ experiences with opioid maintenance treatment and the relation to recovery

**DOI:** 10.3402/qhw.v11.31992

**Published:** 2016-10-21

**Authors:** Trond Erik Grønnestad, Hildegunn Sagvaag

**Affiliations:** Department of Health Studies, University of Stavanger, Stavanger, Norway

**Keywords:** Illicit drug scenes, OMT, stigma, recovery, cheating

## Abstract

**Objective:**

The objective of this article is to gain insight into how individuals who frequent open illicit drug scenes experience opioid maintenance treatment (OMT) and investigate how this appears to affect their recovery processes.

**Method:**

By means of the ethnographic method, one of the researchers spent time in an open illicit drug scene over a 1-year span, and gathered data on individuals who frequent the scene on a regular basis, and their experiences with OMT. The data are based on field notes and audiotaped interviews.

**Findings:**

Four themes emerged as relevant for the participants’ experiences with OMT: 1) the loss of hope, 2) trapped in OMT, 3) substitution treatment is not enough, and 4) stigmatization of identity.

**Conclusion:**

The participants found the OMT to be overruling and degrading. Several of the individuals from the illicit drug scene are part of the OMT programme, but as the treatment does not remove painful emotions, they supplement OMT with illegal substances, violate the OMT regulations, and run the risk of being excluded from the programme. In fear of losing the replacement opioid, they conceal parts of the addiction they seek treatment for and end up lying and cheating instead of exploring strategies for reducing and managing the addiction. The patients’ relation to the OMT personnel is negatively affected by the need to hide a large portion of their issues. The result is a feeling of hopelessness, increased stigmatization, lack of control and being trapped between two worlds—in limbo, an intermediate state which interferes with the recovery process.

In a study of people in an open illicit drug milieu, “the Bench” (Grønnestad & Lalander, 2015), the issue of opioid maintenance treatment (OMT) became significant in a negative way for both persons in OMT treatment and persons outside OMT treatment. Blaming OMT for their own difficult situation became a kind of common adage, so prominently it seemed crucial to investigate in order to understand their experiences. This open drug scene and the people there are the subject of this study: individuals who frequent an open illicit drug scene in a medium-sized Norwegian city and their experiences with OMT.

An open drug scene is defined as “all situations where citizens are publicly confronted with drug use and drug dealing. These scenes vary in visibility, size and site and might be categorized as concentrated open scenes, dispersed open scenes, and hidden scenes” (Waal, Clausen, Gjersing, & Gossop, 2014, p. 1). Unlike large, open illicit drug scenes such one can find in large cities in European like Amsterdam, Frankfurt, Vienna, Zürich and Lisbon's (Waal et al., 2014), the drug scene in this study is not primarily a drug market, but rather a meeting place for people in a medium-sized Norwegian city, who wish to interact with people in the same situation, and to experience a sense of self-worth (Grønnestad & Lalander, 2015). Everyone on the scene uses some form of intoxicant. Some use heroin, many use amphetamine, and practically everyone uses hash, alcohol, and pills. Most of them are part of the OMT programme or have been at some point. They belong to the group of people Gossop (2015) characterize as being “unable to cope with substance abuse.”

In Norway, OMT is part of an overall rehabilitation plan. Patients pick up the medication on average 3.8 times a week and submit urine samples while supervised 0.6 times a week to indicate whether they have taken illicit drugs on the side. Most patients have a permanent place of residence, receive disability benefits, and many suffer from mental illnesses and somatic disorders. Only 48% cope with their addiction to a satisfactory level (Waal, Clausen, Gjersing, & Gossop, 2014). This is in line with British outcome research, which shows that <30–50% of the patients achieve stability by taking the prescribed medication (Gossop, 2015). Even though less than half of the patients cope sufficiently with their addiction, Norwegian studies show that several other OMT goals are reached, drug-induced overdoses were reduced by 80%, overall mortality was reduced by 50% (Clausen, Anchersen, & Waal, 2008), drug-related ill health was reduced by 76% (Skeie et al., 2011), and crime was reduced by 60% (Bukten et al., 2012). Long-term treatment is indicative of reduced drug use, increased social functioning, and an increase in mental well-being (Fingleton, Matheson, & Holland, 2015; Jones, 2007). Interrupted treatment, however, is indicative of increased drug use and somatic issues (Skeie et al., 2013). As it achieves several goals, OMT is thus viewed as a successful method for treating opioid dependence. Some people, nevertheless, feel trapped and unable to handle their addiction (Neale, Pickering, & Nettleton, 2012), or that the treatment causes a new form of dependency that keeps them stuck in a stigmatized position (Järvinen, 2008).

Persons with drug problems, as well as persons with mental disorders, are stigmatized in various forms and that this stigma increases when people have been in treatment and labelled with a diagnosis (Ahern, Stuber, & Galea, 2007; Sirey et al., 2001). High level of secrecy and shame and low degree of psychological flexibility and quality of life are strongly associated with perceived stigma (Luoma et al., 2007), and this effect of stigma persists even after the disease is under control (Link, Cullen, Frank, & Wozniak, 1987). Hughes (1945) described this as “masterstatus” that usually implies a negative connotation related to the negative effects upon an individual being openly labelled as deviant, as the drug user or the mental patient (Becker, 1963; Hughes, 1945). Modified labelling theories show that being labelled with a stigmatized diagnose results in a spoiled identity who is linked to negative outcomes in terms of employment, income, social function, and self-esteem (Wright, Gronfein, & Owens, 2000). This is shown in “Disciplining addiction”, where Philippe Bourgois explains how the system gains power over the patients, making them helpless and where medication and control become the focus of the treatment (Bourgois, 2000). Frida Petersson (2013) asserted that substitution treatment who has become the dominating method for treating substance dependence in Sweden is a system who controls the patients by extensive regulations where the patients are exposed to demeaning inspections and where treatment can be terminated if the rules are violated (Petersson, 2013).

In 2003–2004, the Norwegian Directorate of Health (Helsedirektoratet) carried out an evaluation of OMT. They found that there were large regional differences in practice, where practice often is more influenced by habits and ideology than actual research and also that there was too much focus on the medicinal side of treatment and less on rehabilitation (Helsedirektoratet, 2010). This is not in line with the Norwegian OMT, which consists of two equal pillars: substitute medication and rehabilitation with an individual plan for each patient. The individual plan shall ensure a holistic, coordinated, and individual service where there at any given time is a service provider that has the primary responsibility for the follow-up. The plan should help to clarify the patient's goals, resources, and needs for services. There should be an assessment of measures that can help to cover the need for support (Helsedirektoratet, 2010).

The regulations for OMT, Section 2, state:

The purpose of opioid maintenance treatment is to increase the quality of life for people with substance dependence, and to help them change their situation by improving their ideal level of coping and functioning. The purpose is also to reduce the harmful effects of substance dependence and the risk of drug-induced death. (Helsedirektoratet, 2010)

The national guidelines for OMT stress that the patient's own goals shall be the basis of the treatment, and that OMT shall assist the patient in finding resources that optimize their particular route to recovery (Helsedirektoratet, 2010). User participation and empowerment are presumed to be vital in the process of patients gaining control of their own lives. The goals described in the regulations and guidelines have much in common with the central aspects in a recovery approach. The recovery approach has recently gained momentum within drug addiction treatment in the US, the UK, and several other European countries such as Norway. The approach is included in Norwegian health and welfare policies, for instance, in the guidelines for Concurrent Substance Abuse and Mental Health Disorders (Helsedirektoratet, 2012) and in the guide “Coping together” (Helsedirektoratet, 2014).

Still there is no clear definition of the term recovery, this is a hinder for both clinical practice and research, and it contributes to a variability in reported outcomes of treatment (Laudet, 2007).

The first use of the term recovery in behavioural health goes back to 1935 from the self-help movement in addiction with Alcohol Anonymous and the various abstinence-based or 12-step derivatives as Narcotic Anonymous (Davidson et al., 2005; White, 2000). People who are maintaining abstinence from alcohol or drug use have described themselves as being in recovery (White, 2000). In this tradition, recovery means that the person is no longer using substances but is still vulnerable to relapse and therefore has to protect their sobriety.

Another tradition in drug treatment, which started in Britain in 1926, is harm reduction, the so-called “British system.” Heroin addiction was defined as a disease and legitimized the prescription of injectable heroin on a maintenance basis for people addicted to heroin (Duke et al., 2013). By the mid-1970s, prescribed heroin was displaced with oral methadone (OMT) and in 2004, as much as 1,810,500 persons were prescribed methadone on a maintenance basis in Britain. Research evidence and treatment outcome indicators showed that this praxis was successful in crime reduction but not so helpful in helping people to recover and to sustain drug free (Duke et al., 2013). For several years there was, and still is a discussion between these two perspectives in drug policy and treatment. No one could however dispute that recovery is the ultimate goal for treatment, and therefore recovery became a contest arena, which probably is one reason why it is so difficult to define. Still harm reduction shades into legislation and recovery shades into abstinence (Duke et al., 2013). While recovery in substance treatment often is equivalent to abstinence from substances (recovery from substance dependence), recovery in the mental health field is viewed also as a process, and not just a result (recovery in mental illness) (Davidson & Roe, 2007). This is a relevant division, as one cannot decide to stop a mental illness in the same way as one can stop taking drugs, assuming taking drugs represent the only problem. In this case, recovery constitutes the process of learning to live with the illness and experience well-being despite of it. Although there are several interpretations of recovery, most people agree that recovery-oriented treatment involves helping individuals reach their goals and become active participants in the community (Neale, Nettleton, & Pickering, 2011). Many OMT patients find themselves in a position where they receive replacement opioids (harm reduction) to terminate or reduce the use of illicit substances, while also having to adjust to life in the OMT programme. A large portion of the patients in OMT programme also suffer from mental illnesses and have to learn to live with their mental problems (Clausen et al., 2008; Helsedirektoratet, 2010; Melberg, Lauritzen, & Ravndal, 2003), as well as social problems caused by, or as a part of the reasoning for using heroin. Recovery for persons receiving OMT is therefore a process that is much similar to recovery in mental illness described in Anthony's (1993) definition of recovery as a process:

A deeply personal, unique process of changing one's attitudes, values, feelings, goals, skills, and/or roles. It is a way of living a satisfying, hopeful, and contributing life even with limitations caused by illness. Recovery involves the development of meaning and purpose in one's life as one grows beyond the catastrophic effect of mental illness. (p. 15)

Based on this, we assume that treatment outcome of OMT is recovery (in) also for persons for being stigmatized and identified in an open drug scene. In a systematic review and modified narrative synthesis of the term recovery, five-core elements were identified as important to facilitate the recovery process: 1) empowerment and reclaiming control over one's life, personal ties, 2) hopes and beliefs for the future, 3) rebuilding positive personal identity and overcoming stigmas, 4) finding meaning in life, and 5) connectedness to adores (Leamy et al., 2011).

The objective of this article is to gain insight into how individuals who frequent open illicit drug scenes experience OMT and how this appears to affect their recovery processes.

We therefore ask following questions:

How do individuals who frequent illicit drug scenes experience opioid maintenance treatment?

## Method

To make contact with people on the illicit drug scene one of the researchers—the first author—spent time at the site a few hours a day, 3 days a week over the course of 1 year, and had afterword monthly contact with the users of the drug scene the next 3 years, as part of a larger ethnographic study. It was challenging to become accepted and avoid association with the authorities and the power and control they possess, a common issue when conducting research in marginalized communities (Becker, 1963/1997). To gain access to the research target, the researcher sought out an intercommunal meeting point for persons using illicit substances, over the course of 3 weeks. The service providers at the meeting point were highly regarded in the drug community and functioned as gate openers to the drug users there (Hammersley & Atkinson, 1996/2010). These people later acted as gate openers for the researcher when he entered the drug scene.

### Participants and data collection

The data are based on field notes and audiotaped interviews from people with experiences from OMT programme and the open drug scene. The main field study was carried out between May 2012 and May 2013, while the interviews were conducted between June 2012 and April 2015. The researcher was in contact with 70–80 persons of the users on the drug scene and did get well known with many of them. Five of them was asked to participate in an interview in order to provide a deeper reflection of their life as drug user and their experiences with OMT. In order to gain a gender balance, we expanded the interviews with three women participating in OMT programme, but for the time of interview they were in prison. A total of eight people interviewed using an semi-structured interview guide were asked to tell their story, their experiences with drug and treatment, how it is to be a man or women in the drug scene, and their hope for the future. The interview's lasted from 30 to 90 min. Three of the people on the drug scene are interviewed to times, so a total of 11 interviews. In the field note, we found descriptions from 17 people who talked about their experiences with OMT. In total, our participants consist of 7 women and 18 men with an average age of roughly 40 years. The interviews were audiotaped and transcribed in verbatim. The field notes are written down immediately after the observation describing what happens on the drug scene, how many people, what they do, and what they talk about and also describing the nearest surroundings reaction to the activity on this spot.

### Data analysis

The participants mainly described OMT in negative terms and the concept of stigma became obvious. OMT is a facility for damage reduction, and it is therefore necessary to look into whether the participants’ experiences with OMT interfere with the goal of damage reduction. The balance between damage reduction and damage increase will be interpreted within social theory of stigma in light of elements that facilitates recovery (Leamy et al., 2011).

The data were analysed according to (Lindseth & Nordberg 2004) analysis method with naive reading, thematically structured analysis, units of meaning, subthemes, and main theme. The main theme was confronted with the transcribed text as part of a hermeneutic circle, and recontextualized to achieve an overall understanding (Lindseth & Norberg, 2004).

To enhance the credibility of the study, the analysis has been carried out by two researchers. Both the interviews and the field notes are accounts of people's lives, but the disadvantage of field notes is that even before they are written down, they have been interpreted on the basis of the researcher's preconceptions. To deal with this, the researcher has been in mind that there is no thing as immaculate perception and therefore tried to separate theory from data collection and not drive theory into the data collection process. It has been important to acknowledge the preconceptions in order to challenge them and to be in mind that our interpretation is only one of a possible number of interpretations (Smith, 2015). To handle this, the researcher on the field reported the observation to the other researcher who helped to challenge his preconceptions and to hold insights in mind. To be aware of the “lens behind the eyes” (Gadamer, 2007).

The study is carried out according to the ethical principles put forth by the Declaration of Helsinki (World Medical Association, 2013). The study has been approved by the Norwegian Social Science Data Services (NSD). The participants’ names and characteristics have been altered, the participants have agreed to participate and have been informed that they may withdraw their consent at any time without suffering any consequences. The requirements for anonymity and confidentiality have been met.

## Findings

Four main themes concerning how OMT is experienced by individuals on an open illicit drug scene were discovered. The themes are: 1) the loss of hope, 2) trapped in OMT, 3) substitution treatment is not enough, and 4) stigmatization of identity.

### Loss of hope

The leading motivation for joining the OMT programme and giving up heroin was that the individuals are worn out and fed up with their living conditions and the constant hunt for heroin, as well as longing for a “normal” life. “Arne” (40) has been using heroin since the age of 21 and has lately been taking a gram of heroin every day, just to stay “healthy,” as he puts it. When interviewed a few days before commencing OMT, he says: *I'm fed up. I'm just excited to get into OMT and start taking methadone and have a normal life and start working again*. He is divorced from his wife but is very close to his two sons, who mean the world to him. The need for heroin and the struggle to finance his drug use have been interfering with their relationship, and this has been hard on him. Talking about the anticipated treatment brings a smile to his face.

Two years later, the researcher meets Arne again. He is wearing dirty clothes, has several smaller wounds and infections, and seems to be excessively intoxicated. He tells that he does not share his problems with anyone in the OMT programme, he just gets his daily dose, and leaves. *I'm hooked on methadone and everything else I can get hold of. This is going to kill me soon* (“Arne”). In addition to the methadone, Arne is still using all the drugs he can get hold of. He is no longer expressing any hope for the future, he is staring at the ground with a blank expression on his face.

“Lene” (45) conveys a similar loss of hope. She joined the OMT programme with her partner. While hoping to get her life sorted, she discovered that her partner had “cracked,” taken up heroin, and put them in debt.

I wanted a clear head and to live my life, I had dreams, wanted a family. I had a lot of goals … I then confronted my partner, hey, you're lying to me, I know what's going on, and that it's been going on for a long time. … I was so embarrassed, so ashamed.

Lene sought help from the OMT staff, but did not feel as though she received the understanding and support she needed. She talked to a social worker on the phone but was not offered any further assistance.

Part of the discourse in the drug community focused on being worn out and tired of the constant hunt for heroin and risk of imprisonment. Most of the drug users in this study wanted a change for the better: a stable life with a job, a family, and of course a “clear head” free of intoxication, just like “Lene.” Even though they had struggled with abuse for years, and had seen OMT fail, they expected the methadone to straighten out their lives. When their expectations were not met, or they themselves did not manage to live up to their own expectations their hope was challenged. They then blamed OMT for loosing hope, and expressed that they felt betrayed because the OMT programme offered no assistance in maintaining the hope.

### Trapped in OMT

The participants we were in contact with had been using the replacement opioids methadone or Subutex, or both. When “Lene” began taking methadone, she started craving the substance, and bought pills and illegal methadone in addition to the dose she received from the OMT.

I cracked and took pills when the methadone gave me cravings, and I wanted to tell them that they couldn't give me methadone …. I remember how similar it was to heroin, if I just take a little more it'll feel just like heroin. Today I want to take more to make it feel like heroin, why not? I feel like shit anyway. I stopped doing everything I used to do and sat around at home, so I ended up taking more methadone (additional methadone bought illegally), hoping that it would give me the last finish. But it never did. It's not heroin, you know. (Lene)

Expressed in the above quote, the replacement opioids initially give a high similar to that of heroin, but most users experience withdrawal symptoms if they try to quit. It is in fact so difficult to quit that the Norwegian Directorate of Health advise against it unless the person can manage without opioids. There is a high risk of relapse, overdose, and death when this is not the case (Helsedirektoratet, 2010). The participants feared the withdrawal symptoms of the replacement opioids just as much as they did the heroin withdrawal, except that the replacement withdrawal lasted longer. “Leif” (48) has used heroin for 30 years and has tried several forms of treatment. When the researcher asked him about OMT, Leif reacted with anger, stood up, and started pacing the room. He describes how Subutex traps you:

Subutex makes you ill for 5 months. I'm not kidding—it's insane. If you want to quit, you can't curb it with any pill. There's nothing like it. It's a suffering that can't be described. (Leif)

Most of the participants the researcher talked to had been through several periods of withdrawal, usually because they could not get hold of heroin, but also because they wanted to get clean. They feared the heroin withdrawal, but it was bearable as they knew that the worst symptoms would pass within 1 or 2 weeks. When using replacement opioids, quitting seemed almost impossible.

During the course of the field study, the researcher met several people who used Subutex illegally as their main intoxicant, people who were not part of the OMT programme. To prevent the misuse and leakage of replacement opioids, strict control routines have been introduced for the consumption of opioids and additional drug abuse. One such measure is the regular submission of urine samples to reveal additional drug abuse. The samples must be submitted under supervision, an action which is perceived as a violation of privacy. “Helge” (47):

I get methadone and have to hand in a piss sample twice a week. There's a mirror in front of me so the lady who controls it can make sure I put my penis in the cup. At first it was difficult—degrading, but now I try to laugh it off, which helps. (Field notes)

“Helge” used heroin for many years but is now part of the OMT programme. The fact that he tries to laugh off the supervised urine submission can be seen as a reaction to a degrading situation, an attempt to maintain his dignity. The urine sample must be “clean,” or else he runs the risk of getting his methadone dose reduced or even kicked out of OMT, like “Reidar” (40) describes below:

I had been “clean” for a long time, and felt as though I was getting my life back on track. Then I took a little amphetamine, which showed up in the urine sample, and now they refuse to give me methadone. I don't understand the logic. The methadone is for heroin addiction, and I haven't taken any heroin. The methadone isn't for the amphetamine. (Field notes)

If Reidar is right, this is not entirely in keeping with the OMT regulations, which allow some abuse of illicit substances on the side. Nevertheless, there were several other participants with similar accounts, where what they regarded as “minor slips” brought on major consequences. It may thus seem as though the user's opinion is that OMT practice is stricter than the regulations allow.

Some individuals still had positive experiences with OMT. According to “Nina” (28), she needed someone to supervise her in order to stay off heroin. However, she did not get a say in the size of the Subutex dosage. She received 16 mg Subutex and wanted 8 mg. She was also of the opinion that she could manage without Subutex, it was the aspect of control that kept her from relapsing. Nina needed someone to keep an eye on her. The human contact with someone who sees you was according to her the most important aspect.

The national guidelines for OMT state that the service shall be coordinated between the different authorities the person is in contact with, such as NAV (the Norwegian Labour and Welfare Administration), outpatient clinics, etc. An important part of this is meetings about the patients individual plan. These meetings are meant to assist the patient, but many experience them as another form of control. “Rune” (52) started using heroin after a work place accident left him on disability benefits. He has been part of the OMT programme for several years but lives in constant fear of being kicked out of the programme. A few days prior to a group meeting, he relates that he always dreads these meetings.

I don't experience any support in these meetings, I just get told off. It's a way of making me do what they want. I always bring my mother for support, but everyone doesn't have a mother to take with them. (Field notes)

Rune explains that if he gets kicked out of OMT, he will have to spend his disability benefits on expensive illicit substances, as he does not think he can manage without. If this happens, he would risk missing the mortgage payments on his house and end up on the street. He therefore feels that OMT has great power over him.

The control and demand for abstinence from illicit substances cause some to give up and lose faith in the programme:

I've tried everything to get clean. Rehab institutions, OMT etc., but nothing works. I'm so fed up with the drugs, but I can't handle the OMT routines. There has to be something that works for me? Like in Switzerland, where they hand out heroin as part of the treatment. Then I could have done it. (Finn, 38) (Field notes)

Replacement opioids are addictive in the same way as heroin, but the withdrawal period is longer, which makes it harder to quit (Helsedirektoratet, 2012). The monitoring and inspection to reveal additional abuse is experienced as degrading. The group meetings are seen as another form of control, which is also experienced as degrading. Some claim that they are unable to handle the control aspect, and thus stay away from the programme.

### The notion that substitution treatment is not enough

Another emerging theme was how the replacement opioids did not have the same effect as heroin. “Atle” (42) visits the OMT office a few times a week to pick up methadone. He keeps away from the drug scene and has never really identified with the community. He is freshly shaved, dressed in clean, modern clothes, and stands out from the regular crowd awaiting the distribution. He explains that he was depressed all throughout childhood and shows the scars on his arms from several suicide attempts. He started using hash, amphetamine, and cocaine as a way of self-medicating, but experienced terrible comedowns between the periods of intoxication and gained little from it. While in Oslo to buy drugs, he tried heroin for the first time:

I found myself. I felt a peace and a satisfaction I had never experienced before. After that I was hooked. (Atle)

He used heroin every day for 15 years and financed the consumption by working overtime in his own firm. When the drug abuse was discovered, his wife separated from him and he lost touch with her and the children. He has now been clean for years and has started a new family. One year ago, he tried to resume his job but was unable to because of the painful emotions that returned when he quit heroin, and he now receives disability benefits.

The methadone took away the cravings, but it didn't replace the heroin. I was constantly struggling with severe depression. (Atle)

Many of the people in the community described how nothing made you relax and indifferent to your surroundings like heroin. “Leif,” who has been using heroin for over 30 years, gives the following account:

When you're on heroin, you get all “never mind”—properly “never mind”.

Researcher: Can you get hooked on heroin after one shot?

Leif: Nonsense, that's impossible (laughs). You're not hooked until the nerves kick in. When you wake up in the morning with your nerves on edge—then you're hooked. … You can do heroin for weeks without getting hooked, unless you have something bad in the back of your mind and the shot killed the bad thought. But if you've been fucked as a child and the shot killed the bad thought, you'll be hooked the next day, no question.

The participants have different explanations as to why heroin is so important to them, but a great deal of them use heroin as a coping strategy for painful, traumatic childhood memories, as well as a protection against the guilt they feel about their actions while intoxicated. It seems like heroin is good in cowering the underlying problems who rose to the surface when they stopped using heroin. The replacement opioids did not remove the painful emotions as successfully as the heroin did, and this led many to seek other ways of removing or reducing the pain.

### Stigmatization of identity

Other reasons for staying in the drug community include the loneliness and exclusion many face when attempting to leave and enter ordinary society. “Nina” has four children who are in foster care. She used heroin on occasion in her youth, but stopped when she got married. During her last child birth she was given morphine, which stirred up the old cravings. She then kept her drug use hidden for a long time. While experiencing heroin withdrawal, she was admitted to hospital with a suspected food poisoning, and the addiction was discovered. She was then introduced to OMT. Nina, who is in her mid-30s, describes what it was like to get “clean” with the OMT. She was involuntarily discharged from the programme and now uses any substance she can get hold of. She speaks of her time in the programme:

Suddenly I'm all alone, sober, and my children have been taken from me … The kids, the loneliness, the community that tends to keep its distance because you're a drug addict, that's when stuff happens. Exclusion. … You know what? I'll tell it from a different angle. What they (OMT, other facilities and the child welfare service) do is they lower your expectations to yourself. They don't think an addict can achieve anything, and so that's what happens, because everyone wants to be as comfortable as possible. (Nina)

When family and friends found out about her drug use and the OMT programme, the rumour spread quickly in the neighbourhood. The neighbours and the other mothers at the nursery stayed away from her, even when the children were still in her custody. Her adult siblings stopped giving her lifts, and no one would lend her money. She was no longer included, her surroundings stopped counting on her. This was frustrating to her, but it also allowed her to relax and slack off. The result was that her identity as a wife, neighbour, sister, and mother was exchanged for the identity of a drug addict, and she felt rejected from the community she used to be part of.

Even after being clean for several years, the reactions from the surroundings can complicate the establishment of a new identity. The person quoted below has not been involved with illicit substances for 10 years. She nonetheless has to submit weekly urine samples and pick up her replacement opioids. This is her account of the transition from heroin to OMT:

I think it's because everything is so levelled (when using heroin). We didn't care about anything. And then there's the feeling of being included, you had an identity and respect. When you enter the other life, you're nobody. At first people pat you on the back, but it goes away despite that I've said I need to be seen, or else I'll be lost. I've done it before. If you haven't found your identity, you have to search for it. I'm still searching, I think. (Lene, 45)

When she was on heroin, she didn't care about the reactions from the surroundings, because she was a part of the drug scene where she felt respected and included. She had an identity, she was *someone*. When she joined the OMT programme, she felt as though the things about her that were appreciated in the drug community lost their value, and she became *no one*. To be *no one* was comfortable as it involved lower expectations, but also dangerous because it left her vulnerable. She needed to be supervised by someone in OMT, to be supported, and cheered on in order to refrain from slipping back to the drug scene, as she had done on previous occasions.

Individuals who join the OMT programme have been using heroin for a long time, and the surroundings have assigned them a stigmatized identity with negative qualities that overshadow all the other qualities which also define the person (master status). They also lower their expectations to themselves, a kind of self-stigmatization. This is what “Nina” experienced. Although many of the participants described the drug scene as rotten and deceitful, they still felt included, respected, and to a certain degree appreciated. In ordinary society, they became *no one* and were left vulnerable and at risk of seeking the communities where they were recognized and felt a sense of belonging.

## Discussion

Most of the participants were motivated to join OMT by a combination of being tired of life on the drug scene with its constant hunt for heroin, and wanting a normal life with a home, a job, and a family. Even individuals well into their 40s, who had been addicts almost half their lives, hoped for a normal life when they started on methadone. This hope of a “normal” life appears unrealistic, as the hope has been shattered several times before, and as it is a known fact that integration in the greater society is challenging for people with an extensive substance abuse and connection to the drug scene. Even so, it seems that one clings to the hope of a better life no matter how unrealistic it may be. In an ethnographic study of the drug scene in Malmö, Sweden, Svensson (1996/2007) relates: *The dream is at risk of being shattered when the ex-addict tries to enter the ordinary world* (Svensson, 1996, 2007, p. 378). Weingarten (2010) stated that for the hope to be fulfilled, it must be reasonable. “Hope” is described as follows:

Hope as a verb, as a practice, leads to different activities than hope as a noun. Reasonable hope as a practice, doing reasonable hope, is oriented to here and now, toward actions that will bring people together to work toward a preferred future. (p. 8)

When the replacement opioids did not have the anticipated effect, they compensated with illicit substances, which is in line with the research of Sælør, Ness, Holgersen, and Davidson (2014). It is a little tricky to understand the disappointment expressed by the users, but maybe they also felt fooled by the Norwegian OMT program who expresses (promise) high demands for rehabilitation were the purpose of the treatment is, as mentioned earlier: “to increase the quality of life for people with substance dependence, and to help them change their situation by improving their ideal level of coping and functioning” (Helsedirektoratet, 2010).

When the replacement opioids did not have the effect the participants hoped for, and OMT did not provide sufficient support and help to handle the transition to “normal” life, the hope was lost. And when hope is the main catalyst of the recovery process (Andresen, Oades, & Caputi, 2003), loss of hope may lead to increased substance abuse (Asher & Gask, 2010).

During the years spent in the drug community, the identity and properties of a drug user are formed, and this becomes part of the person's story and habitus (Bourdieu, 1991). For the participants in this study, the introduction to OMT also involved an attempt to change their social identity, where the identity as a drug addict is toned down and upstaged by other identities. Svensson (1996/2007)described this as a marginal phase, where the individual maintains two social identities and belongs to two social worlds that are antagonistic to each other, unable to choose one of them. When abandoning a stigmatized role, the individual must constantly deal with society's reactions and attitudes toward the stigmatized group they previously belonged to (Goffman, 1963/2014). Even when people stopped using heroin and joined the OMT programme, they were still considered to possess the negative qualities (master status) of drug addicts (Becker, 1963; Hughes, 1945). The contrast between the two societies becomes evident, and it becomes difficult to develop a new identity in the drug-free community. “Lene” has managed to live exclusively on OMT medication for 10 years, but is still searching for her identity. Transforming identity is not just about changing our minds, or simply “selecting” somehow voluntarily a new identity; it involves pursuing a new trajectory, new kinds of participation in changing configurations; learning and experiencing new time horizons (Hughes, 2007, p. 689) and to deal with the societies negative reaction toward the person and also the persons lack of social skills. The individuals also often feel shame, have low degree of psychological flexibility, and also grow accustomed to being discredited, and lose the motivation for change, a kind of self-stigmatization (Link, Struening, Rahav, & Nuttbrock, 1997; Luoma et al., 2007; Skog, 2006). The participants in this study blamed OMT for their troubled life, for not understanding how hard it is to change identity. This might be a way of taking care of ones self-esteem, a way of bending the perspective and standing up against the rules in OMT. “The condemned judge the judges” as in Willis (1987/2014). Overcoming stigmas and developing an identity are important to make recovery possible (Anderson & Levy, 2003). Unfortunately, the opposite seems to take place when individuals from the illicit drug community join the OMT programme and try to enter ordinary society.

Quite a few of the individuals we were in contact with used the replacement opioids to keep the withdrawal symptoms in check, and further supplemented with other remedies/substances to endure painful emotions. It is well known that depression among heroin addicts in OMT is highly prevalent (Peles, Schreiber, Naumovsky, & Adelson, 2007; Schreiber, Peles, & Adelson, 2008). Detoxification of OMT patients can bring about an increase in unpleasant memories and emotions, and without coping strategies and psychological support this can result in relapse (Frantzen, 2001/2011; Notley, Blyth, Maskrey, Pinto, & Holland, 2015). The OMT programme (the replacement opioids) was not enough for our informants. The object of the OMT regulations is among other things to regulate the management of OMT medication, as these are potentially dangerous substances that should not be spread to the illicit drug scene. The individuals in question felt that the OMT programme was controlling their lives, so instead of taking their problems and challenges to the OMT personnel, they went to great lengths to deceive them. An example of such manipulation is the use of substances that cannot be detected by the control measures. When a person runs into demands that interfere with his or her values, desires and goals, the conflict can be reduced by changing one's values, desires or goals, or by reducing the importance of what forms the basis of the demand (Emerson, 1962). In this case, both actions are performed. The discomfort of submitting urine samples is reduced through attempting to laugh it off, whereas the significance of the OMT demands are reduced through continued drug abuse. This is a form of cost reduction where some dignity is preserved (Emerson, 1962).

In general, cost reduction is a process involving changes in values (personal, social, economic) which reduces the pains incurred in meeting the demands of a powerful other. (p. 35)

The connection to friends, colleagues, family, and service providers is an important part of the recovery process. When a person has been using drugs for a long time, the connection to people in the “ordinary society” is reduced (Adams, 2008). The relations to service providers thus take on a more important role. But the patients cannot talk to the OMT personnel about potential drug abuse, as this might jeopardize their place in the programme. Consequently, they are unable to talk about their issues, which may prevent a healthy therapeutic relationship. The Norwegian legislation allows some softening-up of the administration of replacement opioids, to secure a better cooperation with the patients (Helsedirektoratet, 2010). However, the number of group meetings has been reduced over the last years, contrary to the governmental regulations (Waal, Clausen, Gjersing, & Gossop, 2014). The goals of the OMT regulations are to increase the quality of life, to assist in changing living conditions, to optimize the level of coping and function, to reduce injuries and drug-induced deaths, as well as to emphasize the patient's own goals, recourses, participation, and empowerment (Helsedirektoratet, 2010). These goals correspond well with the central elements of the recovery process (Leamy et al., 2011), but they have not been observed in this study. The participants see OMT as a system of control that deprives them of their autonomy, where medication (replacement opioids) makes up the entire solution.

## Conclusion

The individuals described in this study are older people who have been using illicit substances for a long time, who were fed up and hoping for a better life when they entered the OMT programme. This hope, however, did not appear to be reasonable as the road to the ordinary society involved a series of challenges they either suppressed or were unaware of. One of the challenges was the exclusion and stigmatization from the society they wished to enter. Another perhaps equally significant challenge was the realization that the replacement opioids did not reduce painful emotions in the same way as heroin. The transition from heroin to replacement medication is a marginalization phase where the patients are vulnerable. The participants in this study did not feel as though they received the help and support they needed, and the road back to the drug scene was short. Even though they resumed the illicit drug use, several of them continued with OMT to prevent withdrawal symptoms. They were afraid to share their problems with the OMT personnel as they were using illicit substances on the side, and they were unable to handle life without drugs. It became a balancing act where they were controlled by the OMT personnel while simultaneously trying to trick them. They found themselves in a position of being trapped between two worlds, stuck in “limbo,” which means “intermediate state” or “paralysis.” Central aspects to facilitate recovery such as hope, identity, overcoming stigmas, relationships, and taking charge of one's life were not apparent in the participants’ accounts of OMT. They described it as a system that kept them in place and prevented them from managing their own lives. Our findings show that the OMT control mechanisms can have negative consequences that overshadow the actual damage reduction. A more flexible and individualized treatment with optional control may perhaps be conductive to an open dialogue and a genuine collaboration on the individual's recovery process, where the goal is not necessarily sobriety, but the experience of moving toward a better life. One way of doing this could be to give persons in OMT program amnesty from illicit drug use. In this way, the health worker could provide OMT treatment in collaboration with the patient and urine sample (if necessary) would only be a subject or a way into a conversation about what it is all about.
